# Factors Affecting the Cost Effectiveness of Antibiotics

**DOI:** 10.1155/2011/249867

**Published:** 2011-02-06

**Authors:** Steven Simoens

**Affiliations:** Research Centre for Pharmaceutical Care and Pharmaco-Economics, Katholieke Universiteit Leuven, Onderwijs en Navorsing 2, P.O. Box 521, Herestraat 49, 3000 Leuven, Belgium

## Abstract

In an era of spiraling health care costs and limited resources, policy makers and health care payers are concerned about the cost effectiveness of antibiotics. The aim of this study is to draw on published economic evaluations with a view to identify and illustrate the factors affecting the cost effectiveness of antibiotic treatment of bacterial infections. The findings indicate that the cost effectiveness of antibiotics is influenced by factors relating to the characteristics and the use of antibiotics (i.e., diagnosis, comparative costs and comparative effectiveness, resistance, patient compliance with treatment, and treatment failure) and by external factors (i.e., funding source, clinical pharmacy interventions, and guideline implementation interventions). Physicians need to take into account these factors when prescribing an antibiotic and assess whether a specific antibiotic treatment adds sufficient value to justify its costs.

## 1. Introduction

Antibiotics have made a significant contribution to improving the health of patients suffering from bacterial infections. For instance, antibiotics are commonly used in the treatment of lower respiratory tract infections. The scientific literature and international guidelines recommend antibiotic therapy in patients with acute exacerbations of chronic obstructive pulmonary disease (COPD) and community-acquired pneumonia (CAP) [1–3]. Also, antibiotics appear effective in improving cure rates and decreasing duration of acute sinusitis in patients who have a microbiological diagnosis of bacterial infection or severe disease [4]. In fact, the added value of antibiotics for therapeutic and prophylactic purposes is so persuasive that many older antibiotics never underwent controlled clinical trials [5]. 

In an era of spiraling health care costs and limited resources, policy makers and health care payers are also concerned about the cost effectiveness of antibiotics. Economic evaluation is a technique that assesses the cost effectiveness of antibiotics by exploring whether antibiotic treatment makes a sufficient contribution to health to justify its costs. An economic evaluation is defined as a comparative analysis of at least two health technologies in terms of both their costs and outcomes [6]. 

Information about the cost effectiveness of antibiotic treatment of bacterial infections can be used for decision-making purposes by a variety of stakeholders [7]. Policy makers can use economic evaluation to inform the allocation of scarce health care resources. Health care payers in an increasing number of countries apply evidence about cost effectiveness to inform drug pricing/reimbursement decisions (see Table 1). Antibiotics that provide better cost effectiveness are rewarded by means of a more favourable price/reimbursement. Health care professionals can rely on economic evaluation to shed light on alternative approaches to treat bacterial infections. Finally, pharmaceutical companies can employ techniques of economic evaluation to demonstrate the cost effectiveness of their antibiotics.

A number of economic evaluations assessing the cost effectiveness of antibiotic treatment of bacterial infections have been published in the literature. The aim of this study is to identify and discuss the factors that affect the cost effectiveness of antibiotics.

## 2. Materials and Methods

The literature review did not focus on presenting evidence about the cost effectiveness of antibiotics but rather drew on published economic evaluations with a view to identify and illustrate the factors affecting the cost effectiveness of antibiotic treatment of bacterial infections. As such, the literature review of economic evaluations was not systematic.

Economic evaluations were identified by searching PubMed, Centre for Reviews and Dissemination databases (Database of Abstracts of Reviews of Effects, National Health Service Economic Evaluation Database, and Health Technology Assessments Database), Cochrane Database of Systematic Reviews, and EconLit up to September 2010. Additionally, the bibliography of included studies was checked for other relevant studies. Search terms related to multiple infection types and antibiotic classes and included “pharmaco-economics,” “economic evaluation,” “cost effectiveness,” “cost minimisation,” “cost utility,” and “cost benefit” alone and in combination with each other. 

The review focused on studies published between 1995 and 2010. Earlier studies were considered to be of limited practical relevance due to likely changes over time in antibiotic treatment modalities and in the organisation and financing of health care systems. Both original economic evaluations and literature reviews of economic evaluations were included.

## 3. Results

**Figure 1:**
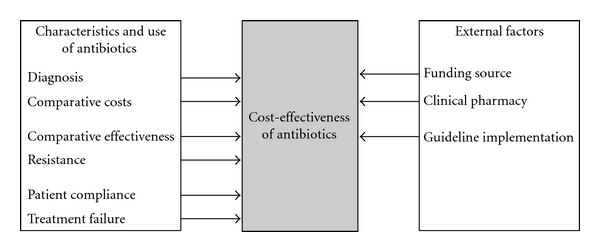
Factors affecting the cost effectiveness of antibiotics.

The cost effectiveness of antibiotic treatment of bacterial infections is influenced by factors relating to the characteristics and the use of antibiotics (i.e., diagnosis, comparative costs and comparative effectiveness, resistance, patient compliance with treatment, and treatment failure) and by external factors (i.e., funding source, clinical pharmacy interventions, and guideline implementation interventions) (see Figure 1).

### 3.1. Diagnosis

Diagnosing a bacterial infection is rendered difficult by the fact that the diagnosis is generally based on patients' self-reported clinical symptoms. This is exemplified with the case of COPD exacerbations. The diagnosis of a COPD exacerbation is complex because exacerbations are heterogeneous and there is debate about the definition of an exacerbation. Furthermore, in practice, high-quality sputum specimens are not always available [8]. This implies that exacerbations are not always identified as such and appropriate treatment is not always administered. In fact, there is evidence that up to 50% of exacerbations are not identified by a health care professional when using a symptom-based definition [9]. 

With respect to the identification of the bacterial aetiology, a Spanish economic evaluation showed that the most valuable treatment strategy for CAP depended on the bacterial pathogen involved and the physician needed to adapt the antibiotic treatment strategy to the cause [10]. The authors concluded that amoxicillin 1 g for treating CAP was more effective and less expensive than moxifloxacin, telithromycin, or clarithromycin if the physician was able to discriminate clinically the bacterial aetiology. If the physician needed to initiate empirical treatment in the absence of information about the causative pathogen and the antibiotic susceptibility pattern of the isolated organism, moxifloxacin became the most valuable option. However, the model of treatment pathways in this study was necessarily simplistic, and future modelling work in this domain would benefit from better and more recent data on resistance.

Viruses can be mistaken for microbial pathogens and may be treated empirically with antibiotics. For instance, two economic evaluations using the same study design explored the cost effectiveness of moxifloxacin in the treatment of CAP in different countries [11, 12]. Viruses were not considered in the base case analysis, and results indicated that moxifloxacin was more effective and less expensive than alternative antibiotics. A sensitivity analysis considered viruses with respect to the prevalence of pathogens; the study assumed a normalized frequency distribution of 20% for viruses, 54% for *S. pneumoniae*, 8% for *H. influenza,* and 18% for atypical pathogens. Antibiotic treatment of pathogens including viruses reduced health care costs, the rate of first-line clinical failure, and the hospitalization rate but did not change the overall conclusions about the cost effectiveness of moxifloxacin. As these economic evaluations were carried out from the perspective of the third-party payer, the analyses considered health care costs only and did not include costs due to productivity loss. The inclusion of indirect costs would result in an even better cost effectiveness for treatment with moxifloxacin.

### 3.2. Comparative Costs and Comparative Effectiveness

The comparative costs and comparative effectiveness of antibiotics play a key role in determining the cost effectiveness of antibiotic treatment of bacterial infections.

A study carried out an economic evaluation of the use of teicoplanin and vancomycin in the treatment of intensive care unit patients with catheter-related infections [13]. Comparative trials of teicoplanin and vancomycin reported no significant differences in their efficacy [14, 15] and, hence, the authors conducted a cost minimisation analysis. In a cost minimisation analysis, only costs are analysed and the least costly treatment approach is chosen because outcomes are known to be equal between approaches. This study elicited data about resource use based on a Delphi panel of nine experts rather than actually observing resource use in patients. Mean treatment costs per patient amounted to 1,272 € with teicoplanin and 1,041 € with vancomycin. The higher treatment cost with teicoplanin mainly originated from higher drug acquisition costs. Treatment costs of teicoplanin and vancomycin turned out to be sensitive to changes in drug unit costs and unit costs of serum level monitoring tests. 

A literature review of antibiotic treatment of COPD exacerbations focused on the comparative costs and the comparative effectiveness of first-generation antibiotics (aminopenicillins, macrolides, and tetracyclines) and second-generation antibiotics (e.g., fluoroquinolones) [16]. Fluoroquinolones generally had higher acquisition costs than first-generation antibiotics. Traditionally, studies suggested that second-generation macrolides and fluoroquinolones are equally effective as first-generation antibiotics [17]. If this is the case, the cost effectiveness of antibiotic treatment can be determined by means of a cost minimisation analysis. However, this literature was limited by the fact that most trials were powered to demonstrate equivalence rather than clinical superiority, had enrolled small samples that are not always representative of the patient population, and did not control for concomitant therapy or for comorbidities. Also, more recent evidence suggested that management of COPD exacerbations with moxifloxacin or gemifloxacin is associated with a shorter time to resolution of symptoms, a lower hospitalisation rate, and a prolonged exacerbation-free interval, thereby generating clinical benefits as well as cost savings [18, 19]. In general, there is a need for economic evaluations to determine the cost effectiveness of treating COPD exacerbations by comparing the comparative costs of antibiotics with their comparative effectiveness.

### 3.3. Resistance

When antibiotics first became available, changes in the susceptibility of pathogens were of little concern. However, inappropriate use of antibiotics, (human-to-human) clonal spread of multidrug-resistant strains, and the presence of comorbidities have all contributed to the rise in resistance over the years. Resistance to antibiotics can have a substantial impact on outcomes and costs of treatment. For instance, there is evidence that CAP patients with pneumococcal resistance may be at greater risk of poor outcomes [20]. Also, if first-line treatment fails due to resistance, additional costs are incurred due to the need for second-line treatment or hospitalization, or both.

Using evidence from four economic evaluations of antibiotic treatment of mild-to-moderate CAP in Belgium, Canada, France, Spain, and the United States [10–12, 21], it is possible to examine the impact of resistance on the cost effectiveness of antibiotics. The studies employed a similar study design; decision-analytic models evaluated the cost effectiveness of oral antibiotics from the third-party payer perspective, with first-line treatment being initiated in the community and failure resulting in second-line treatment in the community or hospitalization. The first-line intervention was moxifloxacin in each study. Comparator treatments were beta-lactams (e.g., coamoxiclav, cefuroxime), macrolides (e.g., clarithromycin, azithromycin), or tetracyclines (e.g., doxycycline). Effectiveness was assessed in terms of the rate of first-line clinical failures, of second-line treatments required, of hospitalizations required, and of mortality.

The impact of resistance on the cost effectiveness of antibiotics was investigated in two ways. First, sensitivity analyses examined the impact of various resistance rates for *S. pneumoniae* and *H. influenzae* on the cost effectiveness of antibiotics. Second, results on cost effectiveness can be compared between economic evaluations and thus between countries with different levels of resistance; Germany has a low level of resistance in CAP pathogens [22]; Belgium, Canada, and the United States have an intermediate level of resistance [23–25]; France and Spain have a high level of resistance [22]. However, it should be noted that factors other than resistance may explain differences in results between these economic evaluations (e.g., costs of care, treatment protocols).

The sensitivity analyses and the comparison between countries indicated that varying levels of resistance in CAP pathogens and multidrug resistance in *S. pneumoniae* isolates affected costs and clinical outcomes of antibiotic treatment [10–12]. However, conclusions did not change; treatment of CAP with moxifloxacin was more effective and less expensive than other antibiotic treatment strategies in Belgium, France, Germany, Spain, and the United States. At the moment, worldwide resistance of CAP pathogens to moxifloxacin is low [26] but continued vigilance with regard to the evolution of resistance and its impact on the cost effectiveness of moxifloxacin and of other antibiotics is indicated.

In Canada, the sensitivity analysis showed that a 50% increase in fluoroquinolone resistance would decrease the cost effectiveness of moxifloxacin treatment as compared with azithromycin to CAN$ 101.47 per first-line clinical failure avoided [21]. Canada has faced a steady increase in macrolide resistance in *S. pneumoniae* over time [23], and further increases in macrolide resistance rates cannot be ruled out. Increases in macrolide resistance would improve the cost effectiveness of treatment with moxifloxacin.

### 3.4. Patient Compliance

The cost effectiveness of antibiotic treatment also depends on patient compliance, with compliance being affected by the frequency of dosing, duration of treatment, adverse events, ease of administering drugs, ease of packaging, and price [27]. An economic evaluation of antibiotic treatment quantified patient compliance; rates of compliance defined as an intake of at least 80% of the prescribed dose varied between 76% and 83% [28]. Various strategies to enhance patient compliance with antibiotic treatment have been proposed such as patient education, once-daily dosing schedules, a convenient and acceptable form of medication, easy-to-open packaging, and the choice of an antibiotic with few side effects [27].

### 3.5. Treatment Failure

Resistance to antibiotics and patient compliance may influence the cost effectiveness of antibiotics because they may lead to treatment failure and further antibiotic treatment or hospitalisation. For instance, a literature review of the distribution of health care costs of COPD exacerbations found that hospitalization costs accounted for more than 45% of health care costs and drugs costs made up between 6% and 21% of costs [16]. As hospitalization is generally indicative of treatment failure, these estimates highlight the cost effectiveness that can be attained from more effective antibiotics that allow patients to be managed in primary care and that prevent treatment failure and hospitalization. In other words, if a new antibiotic would have a lower failure rate than alternative treatments, it would be likely to be cost effective, even if it is more expensive than other antibiotics.

Treatment failure may be caused by a number of host factors. The literature suggests that frequency of exacerbations, presence of comorbidities, impairment in lung function, need for more aggressive bronchodilator therapy, and previous hospitalization predict treatment failure [29, 30]. The ability to identify patients at a higher risk of failing treatment can aid clinicians in their choice of antibiotic. This implies that it may be advisable to identify patient subgroups in which treatment with a specific antibiotic provides the best cost effectiveness and should be recommended by guidelines.

### 3.6. Funding Source

A recent study extracted the cost effectiveness of antibiotics from economic evaluations included in the Tufts-New England Center Cost Effectiveness Analysis Registry through September 2009 [31]. The analysis included 85 observations on the cost effectiveness of antibiotics derived from 23 economic evaluations. Economic evaluations related to infectious diseases (58% of studies), respiratory diseases (13%), cardiovascular diseases (9%), critical care (4%), endocrine disorders (4%), genitor-urinary diseases (4%), musculoskeletal and rheumatologic diseases (4%), and sensory organ diseases (4%). The results indicated that the median incremental cost effectiveness ratio of antibiotics was 748 € per quality-adjusted life year. Specifically, 38.8% of antibiotics were more effective and less costly than the comparator; 45.9% of antibiotics improved effectiveness but also increased costs; 15.3% of antibiotics were less effective and more costly than the comparator.

The cost effectiveness of antibiotics derived from analyses funded by industry tended to be better than the cost effectiveness derived from analyses funded from other sources (e.g., government, foundations). However, the limited number of observations implied that it was not possible to statistically test for this association. Also, there were too few observations to explore whether there was an association between the methodological quality of economic evaluations and the funding source. The possible association between cost effectiveness and funding source may have several explanations; industry influences the design of economic evaluations with a view to improving the cost effectiveness of their products; as costs of research and development are high, industry markets those antibiotics that are cost effective only; industry sponsors economic evaluations of antibiotics that are likely to be cost effective only; researchers conduct and journal editors publish those economic evaluations that support the cost effectiveness of antibiotics. In response to the possible manipulation of studies, professional societies and health care payers are increasingly issuing guidelines for the conduct and reporting of economic evaluations.

### 3.7. Clinical Pharmacy

During the last decades, clinical pharmacy services have developed around the world [32]. Even if there exists no consensus concerning the term “clinical pharmacy,” clinical pharmacy can be defined as the contribution of pharmacists and their assistants to drug therapy as a part of the total care supplied to patients, in cooperation with physicians and nursing staff, with a view to optimizing the cost effectiveness, the effectiveness, and the safety of drug therapy.

A literature review examined the cost effectiveness of clinical pharmacy interventions focusing on the management of antibiotic therapy in a hospital setting [33]. Extracting evidence from six economic evaluations, the authors concluded that clinical pharmacy interventions relating to antibiotic therapy can lower costs of hospital care without adversely affecting clinical outcomes. Lower costs arose from a decrease in drug costs (e.g., due to switch from intravenous to oral drugs), lower pharmacy costs, and a decrease in length of stay. However, economic evaluations of clinical pharmacy interventions suffered from a number of methodological limitations relating to the absence of a control group without clinical pharmacy interventions; limited scope of costs and outcomes; focus on direct healthcare costs only; exclusion of pharmacist employment cost; use of intermediate outcome measures; exclusion of health benefits; absence of incremental analysis.

### 3.8. Guideline Implementation

Numerous guidelines have been published governing appropriate antibiotic treatment of bacterial infections. Interventions surrounding the implementation of guidelines may have an impact on health care professional compliance with guidelines and, hence, may influence the cost effectiveness of antibiotics. 

A literature review evaluated the cost effectiveness of antibiotic treatment consistent with guidelines for patients with CAP [34]. This literature indicated that antibiotic treatment consistent with guidelines reduced length of stay, decreased costs, and reduced the mortality rate. However, existing studies suffered from methodological limitations, and high-quality economic evaluations examining the impact of guideline implementation interventions on the cost effectiveness of antibiotic treatment are needed.

## 4. Conclusions

This study has identified and discussed the factors that affect the cost effectiveness of antibiotics. The findings indicate that the cost effectiveness of antibiotics is influenced by factors relating to the characteristics and the use of antibiotics (i.e., diagnosis, comparative costs and comparative effectiveness, resistance, patient compliance with treatment, and treatment failure) and by external factors (i.e., funding source, clinical pharmacy interventions, and guideline implementation interventions). Physicians need to take into account these factors when prescribing an antibiotic and assess whether a specific antibiotic treatment adds sufficient value to justify its costs. Finally, it should be noted that cost effectiveness is only one of the factors and not necessarily the most important factor informing the choice of physicians between antibiotics. Other factors that need to be taken into account include, for example, route of administration, patient profile, and the occurrence of adverse events.

## Figures and Tables

**Table 1:** Use of economic evaluation in drug pricing/reimbursement.

Country	Organisation	Implementation date
Australia	Pharmaceutical Benefits Advisory Committee	1993
Belgium	Medicine Reimbursement Committee	2002
England and Wales	National Institute for Health and Clinical Excellence	1999
France	High Health Authority	2008
Germany	Institute for Quality and Efficiency in Health Care	2007
Netherlands	Health Care Insurance Board	1999
New Zealand	Pharmaceutical Management Agency	1993
Scotland	Scottish Medicines Consortium	2002
Sweden	Dental and Pharmaceutical Benefits Agency	2002
Taiwan	Centre for Drug Evaluation	2008
